# Changing school start times: Impact on extracurricular activities and employment

**DOI:** 10.3389/frsle.2022.1044457

**Published:** 2022-11-02

**Authors:** Lisa J. Meltzer, Amy E. Plog, Kyla L. Wahlstrom, Janise McNally

**Affiliations:** ^1^Department of Pediatrics, National Jewish Health, Denver, CO, United States; ^2^Wellness Department, Cherry Creek School District, Greenwood Village, CO, United States; ^3^Department of Organizational Leadership, Policy, and Development, University of Minnesota, Minneapolis, MN, United States

**Keywords:** school start time policy, student sleep, elementary school, middle school, high school

## Abstract

Sufficient sleep duration is associated with student health and wellbeing, but early secondary school start times limit students' sleep opportunity. Despite recommendations that all middle and high schools adopt a healthy school start time policy, one barrier to policy implementation is concerns about the impact on student participation in activities and employment. This study examined student extracurricular activity participation and employment before and after the implementation of healthy secondary school start times. Approximately 24,000 students/year (grades 3–11) completed three annual surveys (pre-change, post-change, follow-up) measuring sleep-wake patterns, extracurricular activity participation, and employment. Following the implementation of an earlier school start time, before-school activity participation decreased for elementary school students, but after-school participation was similar across years. Following the implementation of later school start times, there was a small decrease in after-school activity participation for middle and high school students (~3–4%). Equally important, middle and high school students reported significantly increased sleep duration with later start times, regardless of participation in before- or after-school activities and employment. Study findings support the recommendation for healthy school start time policies.

## Introduction

Healthy school start times (no earlier than 8:30 A.M. for middle and high schools) increase sleep duration for secondary students, with other noted benefits including improved attendance and mood, and decreased tardiness, sleepiness, motor vehicle crashes, and caffeine use (Wahlstrom, 2020). However, systemic barriers remain that prevent school districts in the United States from changing school start times, including concerns about extracurricular activity participation and adolescent employment (Owens et al., 2014; Fitzpatrick et al., 2020).

Non-athletic extracurricular activities (e.g., clubs, music, religious/cultural school or groups, volunteering) and part-time employment (<20 h/week) have been shown to be associated with positive outcomes for students, including improved academic achievement and prosocial behavior (Mortimer, 2003; Farb and Matjasko, 2012; Tjaden et al., 2019). However, participation in athletics, extracurricular activities and employment may negatively impact adolescent sleep duration (Owens, 2014), delaying bedtimes or requiring earlier wake times.

In order to address the ongoing concern about how participation in extracurricular activities and employment may be impacted by a change in school start times, and how participation in activities and employment may impact sleep, this study evaluated non-athletic extracurricular activity participation, employment, and sleep before and after implementation of new start times for elementary, middle, and high school students. The study was designed to address three specific gaps within the literature. First, although several studies have demonstrated that later school start times do not impact after-school participation in extracurricular activities or employment (Wahlstrom et al., 2014), this study considered how changing school start times impacted both before- and after-school participation. Second, the study examined whether sleep-wake patterns differed based on student participation in before- and after-school extracurricular activities or employment, and whether sleep duration is associated with those endeavors after implementation of new start times. Third, this is the first study, to our knowledge, to examine the impact of earlier start times on elementary school student participation in before- and after-school extracurricular activities. This is critical because elementary schools are often required to start earlier when secondary school start times are delayed, yet very few studies have examined the impact of school start time policies on elementary school students. It is important to note that this paper does not seek to compare changes in sleep-wake patterns across years, as these data have previously been published (Meltzer et al., 2021, 2022).

## Methods

### Procedure

The Cherry Creek School District (CCSD) changed school start times for the 2017–2018 school year, with high schools [HS] delayed 70 min (from 7:10 am to 8:20 a.m.), middle schools [MS] delayed 40–60 min (from 7:50–8:10 am to 8:50 am), and elementary schools [ES] advanced 1 h (from 9:00 am to 8:00 am). Students in grades 3–11 completed online surveys during school hours that assessed sleep-wake patterns (i.e., weekday bedtime, wake time, sleep duration) and participation in extracurricular activities (defined as “e.g., dance, music lessons, religious/culture school, volunteer, etc.”) before and after school. HS students were also asked about employment before and after school. For ES students, survey questions and responses were read aloud if needed. Surveys were administered in three waves: ~4 months prior to the start time change (pre-change, *n* = 21,520), ~6 months (post-change, *n* = 23,794), and ~18 months (follow-up, *n* = 26,925) after the start time change. Surveys were anonymous and the study was approved by the CCSD's Research Review Committee.

### Participants

Supplementary Table S1 provides complete demographic information for participants by level (ES, MS, HS) across the 3 years of the study. Overall, the study sample was representative of the CCSD in terms of race/ethnicity and gender: 49.8% female, 55.1% White, 10.7% Black, 17.8% Hispanic, and 8.9% Asian (CCSD is 50.5% White, 11.4% Black, 20.9% Hispanic, 8.6% Asian), but less representative of students who qualified for free/reduced lunch (20.7% in the study vs. 29.0% in the CCSD).

### Data analyses

As surveys were anonymous data could not be linked longitudinally. All analyses were conducted separately for school level (ES, MS, HS). Chi-square analyses were used to compare the percent of students who participated in activities and employment across years. Welch's ANOVA (due to unequal sample sizes) was used to compare sleep-wake patterns for students who did and did not participate in before- and after-school activities or employment separately for each year of the study. Chi-square analyses were also used to examine the percent of students obtaining sufficient sleep duration (defined as >9 h for ES/MS, >8 h for HS) (Office of Disease Prevention and Health Promotion, 2020) across years. Due to the large sample size and the number of comparisons, statistical significance was conservatively set at *p* < 0.001, and effect sizes were used to examine the magnitude of difference (Cramer's V for chi-square analyses [small = 0.07, medium = 0.21, large = 0.35] and Cohen's d for ANOVA analyses [small = 0.2, medium = 0.5, large = 0.8]) (Cohen, 1988).

## Results

### Participation in activities and employment

**Figure 1:**
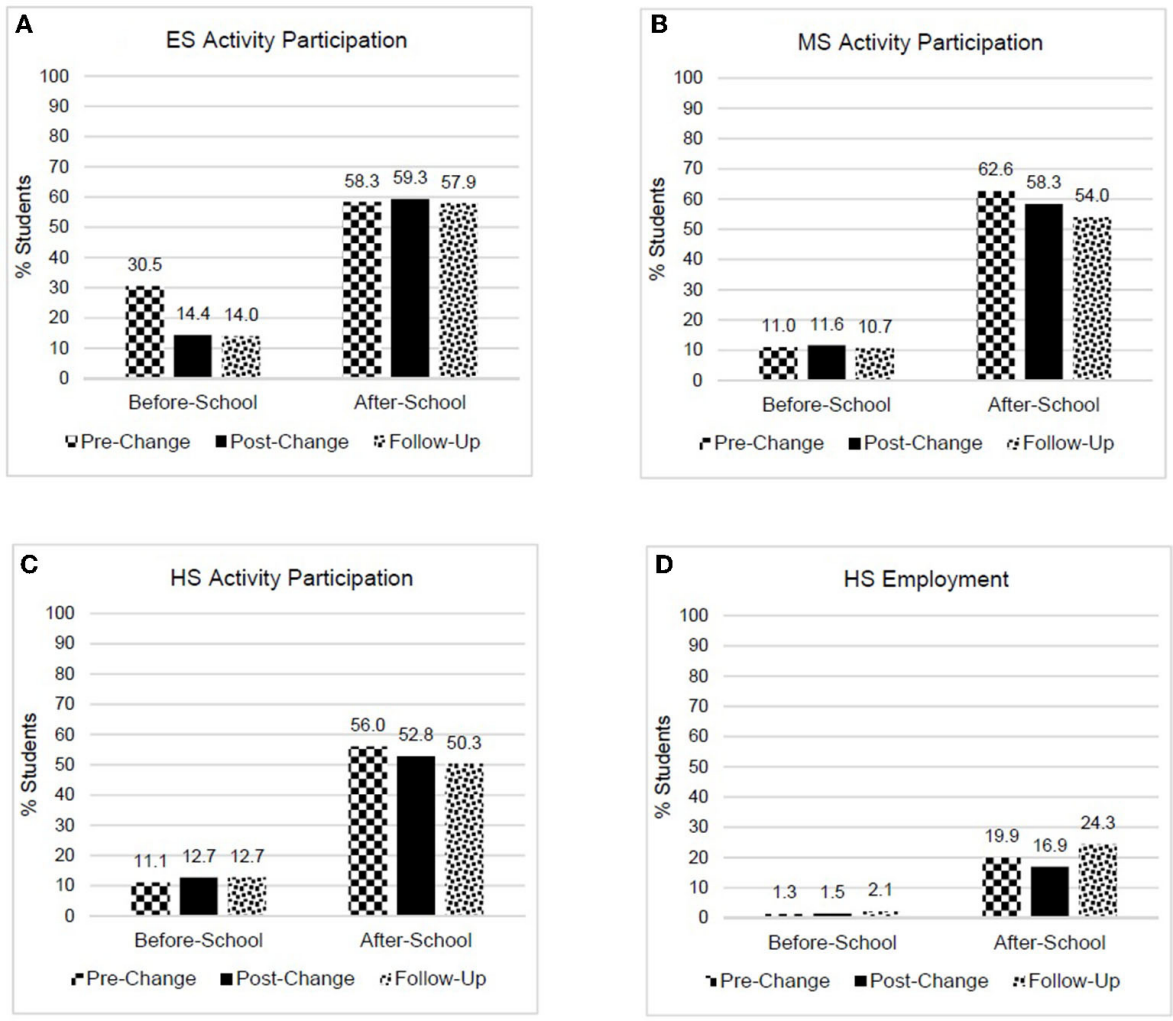
Percent of students participating in before- and after-school activities or employment by year. **(A)** Elementary school activity participation, **(B)** middle school activity participation, **(C)** high school activity participation, and **(D)** high school employment.

After changing to earlier start times, significantly fewer ES students participated in before-school activities, *X*^2^(2) = 920.56, *V* = 0.19 (small effect size, Table 1), while no change was found for the percent of ES students who participated in after-school activities, *X*^2^(2) = 3.55, *V* = 0.01 (Table 1 and Figure 1A). After changing to later start times, there was no notable change in the percent of MS students, *X*^2^(2) = 3.53, *V* = 0.01, or HS students, *X*^2^(2) = 10.11, *V* = 0.02, who participated in before-school activities (Table 1 and Figures 1B,C). The percent of students participating in after-school activities was lower for MS students, *X*^2^(2) = 124.18, *V* = 0.07 (small effect size, Table 1 and Figure 1B). Although the percent of HS students participating in after-school activities was also lower, the effect size was not meaningful, *X*^2^(2) = 47.69, *V* = 0.05 (Table 1 and Figure 1C). The percent of HS students who were employed before-school did not change, *X*^2^(2) = 16.10, *V* = 0.03, while the percent of HS students who were employed after-school was initially lower at post-change compared to pre-change, but was higher at follow-up, compared to both pre-change and post-change, *X*^2^(2) = 138.07, *V* = 0.08 (small effect size, Table 1 and Figure 1D). The percent of students who participated within levels and across years was similar across demographic characteristics (Supplementary Tables S2, S3). However, the percent of students who reported after-school employment did not change from pre-change to post-change for students who qualified for FRL, but was 3.5% lower for non-FRL students. Further, for FRL students, after-school employment was ~10% higher at follow-up compared to post-change, with after-school employment among non-FRL students ~7% higher (Supplementary Table 3).

**Table 1:** Comparison of sleep-wake patterns by participation in extracurricular activities or employment at each survey time point [data are presented as mean (SD) unless otherwise indicated].

	**Before-school participation**					**After-school participation**			
	**No**	**Yes**	***F/X^2^***	***d/V***		**No**	**Yes**	***F/X^2^***	***d/V***
**Elementary school extracurricular activities**									
Participants [n (%)]			920.56^*^	0.19				3.55	0.01
Pre-Change	5,806 (69.5%)	2,543 (30.5%)				3,478 (41.7%)	4,871 (58.3%)		
Post-Change	6,801 (85.6%)	1,142 (14.4%)				3,231 (40.7%)	4,712 (59.3%)		
Follow-Up	7,096 (86.0%)	1,157 (14.0%)				3,475 (42.1%)	4,778 (57.9%)		
Bedtime									
Pre-Change	21:12 (0:57)	21:13 (0:59)	0.02	0.00		21:13 (1:00)	21:12 (0:56)	0.06	0.01
Post-Change	21:00 (0:56)	21:06 (1:07)	8.19	0.10		21:03 (1:01)	20:59 (0:55)	6.72	0.06
Follow-Up	21:01 (0:57)	21:05 (1:07)	3.11	0.06		21:05 (1:00)	20:59 (0:57)	16.10^*^	0.09
Wake time									
Pre-Change	7:05 (0:49)	7:04 (0:55)	1.19	0.03		7:03 (0:51)	7:06 (0:50)	5.14	0.04
Post-Change	6:42 (0:45)	6:44 (0:55)	1.83	0.05		6:42 (0:49)	6:43 (0:44)	0.79	0.02
Follow-Up	6:41 (0:45)	6:45 (0:57)	4.64	0.07		6:41 (0:48)	6:42 (0:46)	1.12	0.02
Sleep duration (hrs)									
Pre-Change	9.88 (1.10)	9.85 (1.17)	0.89	0.03		9.84 (1.15)	9.89 (1.01)	3.61	0.06
Post-Change	9.70 (1.04)	9.64 (1.22)	2.51	0.05		9.65 (1.11)	9.72 (1.04)	8.92	0.07
Follow-Up	9.67 (1.04)	9.67 (1.25)	0.00	0.00		9.61 (1.10)	9.71 (1.06)	19.40^*^	0.10
% sufficient sleep									
Pre-Change	84.2%	82.0%	6.42	0.03		82.1%	84.5%	8.48	0.03
Post-Change	81.9%	77.3%	13.36^*^	0.04		78.9%	82.8%	18.99^*^	0.05
Follow-Up	80.9%	77.0%	9.42	0.03		78.3%	81.8%	15.69^*^	0.04
**Middle school extracurricular activities**									
Participants [n (%)]			3.53	0.01				124.18^*^	0.07
Pre-Change	6,705 (89.0%)	826 (11.0%)				2,814 (37.4%)	4,717 (62.6%)		
Post-Change	7,460 (88.4%)	975 (11.6%)				3,515 (41.7%)	4,920 (58.3%)		
Follow-Up	7,940 (89.3%)	949 (10.7%)				4,086 (46.0%)	4,803 (54.0%)		
Bedtime									
Pre-Change	21:49 (0:57)	21:50 (1:01)	0.02	0.00		21:51 (1:00)	21:48 (0:55)	2.72	0.04
Post-Change	21:58 (1:01)	21:58 (1:03)	0.13	0.01		21:59 (1:03)	21:58 (1:00)	1.43	0.03
Follow-Up	21:58 (1:01)	21:59 (1:05)	0.15	0.01		22:00 (1:04)	21:57 (0:59)	4.30	0.04
Wake time									
Pre-Change	6:27 (0:38)	6:26 (0:48)	0.46	0.03		6:27 (0:41)	6:27 (0:38)	0.02	0.00
Post-Change	7:05 (0:41)	6:59 (0:45)	15.38^*^	0.14		7:06 (0:43)	7:04 (0:40)	4.88	0.05
Follow-Up	7:03 (0:41)	6:56 (0:47)	22.03^*^	0.17		7:03 (0:43)	7:02 (0:41)	0.89	0.02
Sleep duration (hrs)									
Pre-Change	8.63 (1.01)	8.60 (1.16)	0.33	0.02		8.60 (1.06)	8.64 (1.00)	2.14	0.05
Post-Change	9.11 (1.06)	9.03 (1.14)	4.91	0.08		9.11 (1.10)	9.10 (1.05)	0.08	0.01
Follow-Up	9.08 (1.07)	8.94 (1.18)	12.27^*^	0.12		9.05 (1.12)	9.08 (1.05)	1.84	0.03
% sufficient sleep									
Pre-Change	40.5%	40.4%	0.001	0.00		40.5%	40.4%	0.003	0.001
Post-Change	61.0%	57.5%	4.21	0.02		60.2%	60.8%	0.38	0.007
Follow-Up	60.3%	53.4%	16.77^*^	0.04		58.7%	60.3%	2.42	0.02
**High school extracurricular activities**									
Participants [n (%)]			10.11	0.02				47.69^*^	0.05
Pre-Change	5,012 (88.9%)	628 (11.1%)				2,481 (44.0%)	3,159 (56.0%)		
Post-Change	6,472 (87.3%)	944 (12.7%)				3,498 (47.2%)	3,918 (52.8%)		
Follow-Up	8,536 (87.3%)	1,247 (12.7%)				4,865 (49.7%)	4,918 (50.3%)		
Bedtime									
Pre-Change	22:23 (0:58)	22:21 (0:59)	0.56	0.03		22:21 (0:59)	22:25 (0:58)	6.20	0.07
Post-Change	22:35 (1:03)	22:33 (1:01)	0.52	0.02		22:33 (1:04)	22:36 (1:01)	5.78	0.06
Follow-Up	22:46 (1:04)	22:43 (1:05)	1.54	0.04		22:45 (1:06)	22:47 (1:04)	2.76	0.03
Wake time									
Pre-Change	5:46 (0:40)	5:46 (0:53)	1.91	0.00		5:47 (0:43)	5:45 (0:41)	4.77	0.04
Post-Change	6:45 (0:39)	6:35 (0:47)	44.87^*^	0.25		6:46 (0:41)	6:42 (0:40)	17.62^*^	0.10
Follow-Up	6:46 (0:41)	6:36 (0:49)	45.71^*^	0.22		6:47 (0:43)	6:43 (0:41)	14.42^*^	0.08
Sleep duration (hrs)									
Pre-Change	7.39 (1.06)	7.37 (1.19)	0.14	0.02		7.44 (1.08)	7.34 (1.07)	13.50^*^	0.14
Post-Change	8.17 (1.11)	8.02 (1.15)	14.89^*^	0.14		8.22 (1.13)	8.09 (1.09)	23.05^*^	0.11
Follow-Up	8.01 (1.12)	7.88 (1.16)	12.14^*^	0.11		8.03 (1.14)	7.94 (1.11)	15.78^*^	0.08
% sufficient sleep									
Pre-Change	30.7%	28.0%	1.90	0.02		32.5%	28.8%	9.05	0.04
Post-Change	64.5%	56.3%	24.33^*^	0.06		66.0%	61.2%	17.99^*^	0.05
Follow-Up	58.4%	52.4%	16.48^*^	0.04		59.4%	56.0%	11.90	0.04
**High school employment**									
Participants [n (%)]			16.10^*^	0.03				138.07^*^	0.08
Pre-Change	5,570 (98.7%)	74 (1.3%)				4,523 (80.1%)	1,121 (19.9%)		
Post-Change	6,956 (98.5%)	105 (1.5%)				5,866 (83.1%)	1,195 (16.9%)		
Follow-Up	9,578 (97.9%)	205 (2.1%)				7,410 (75.7%)	2,373 (24.3%)		
Bedtime									
Pre-Change	22:23 (0:59)	22:25 (1:03)	0.11	0.04		22:22 (0:58)	22:28 (0:56)	12.75^*^	0.12
Post-Change	22:36 (1:02)	22:31 (1:14)	0.53	0.08		22:34 (1:02)	22:45 (1:03)	27.20^*^	
Follow-Up	22:45 (1:04)	22:57 (1:24)	3.58	0.15		22:42 (1:04)	22:58 (1:05)	109.20^*^	0.25
Wake time									
Pre-Change	5:46 (0:41)	6:01 (1:09)	3.33	0.26		5:46 (0:41)	5:47 (0:44)	1.54	0.03
Post-Change	6:43 (0:40)	6:44 (0:57)	0.04	0.02		6:42 (0:39)	6:46 (0:44)	7.76	
Follow-Up	6:45 (0:42)	6:49 (0:57)	1.20	0.09		6:43 (0:41)	6:50 (0:44)	45.34^*^	0.16
Sleep duration (hrs)									
Pre-Change	7.38 (1.07)	7.59 (1.38)	1.62	0.17		7.40 (1.08)	7.32 (1.08)	5.20	0.11
Post-Change	8.11 (1.09)	8.22 (1.45)	0.56	0.08		8.13 (1.09)	8.02 (1.14)	9.51	
Follow-Up	7.99 (1.11)	7.88 (1.52)	1.12	0.08		8.03 (1.11)	7.87 (1.16)	31.56^*^	0.13
% sufficient sleep									
Pre-Change	30.3%	36.5%	1.32	0.02		31.3%	26.8%	8.69	0.04
Post-Change	62.6%	61.9%	0.02	0.002		63.3%	58.8%	8.66	0.04
Follow-Up	57.8%	50.7%	4.12	0.02		58.8%	54.0%	16.97^*^	0.04

* p < 0.001; Cohen's d effect size: small = 0.20, medium = 0.50, large = 0.80, Cramer's V effect size: small = 0.07, medium = 0.21, large = 0.35.

### Sleep-wake patterns and participation in activities or employment

**Figure 2:**
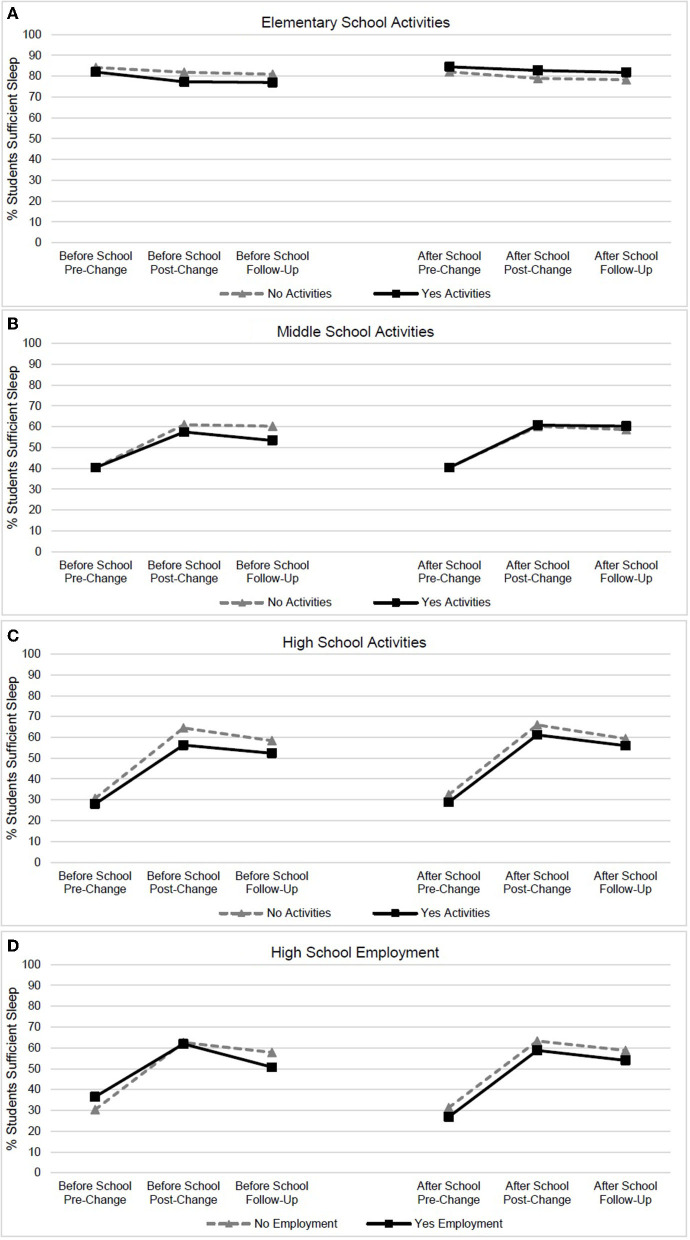
Percent of students obtaining sufficient sleep duration across years for students who do and do not participate in activities/employment (>9 hours for elementary and middle school students, >8 hours for high school students). **(A)** Elementary school activity participation, **(B)** middle school activity participation, **(C)** high school activity participation, and **(D)** high school employment.

Sleep-wake patterns did not differ between ES students who did and did not participate in before-school activities (Table 1). However, at post-change, a lower percent of students who participated in before-school activities obtained sufficient sleep (Table 1 and Figure 2A) compared to pre-change, although effect sizes were not meaningful. At follow-up only, ES students who participated in after-school activities had earlier bedtimes (6 min) and longer sleep duration (6 min), although effect sizes were not meaningful. Similarly, at both post-change and follow-up, a higher percent of ES students who participated in after-school activities obtained sufficient sleep duration (3.5%), although again, effect sizes were not meaningful.

MS students who participated in before-school activities had earlier wake times at post-change (6 min) and at follow-up (7 min) than non-participating students, but effect sizes were not meaningful (Table 1). Sleep-wake patterns and the percent of students obtaining sufficient sleep were similar between MS students who did and those who did not participate in after-school activities across years (Figure 2B).

At post-change and follow-up, HS students who participated in before-school activities had earlier wake times (10 and 9 min earlier, respectively), and shorter sleep duration (9 and 8 min shorter, respectively), than non-participating students; however, a small effect size (*d* = 0.25 and *d* = *0.2*2, respectively), was found only for wake times (Table 1). Similarly, at both post-change and follow-up, a higher percentage of students obtained sufficient sleep duration in the non-activity group (8.2% and 6.0% higher respectively, Figure 2C). HS students who participated in after-school activities had later wake times at both post-change and follow-up (6 min), and a shorter sleep duration at all three time points (6, 8, and 6 min, respectively), than non-participating students, although effect sizes were not meaningful. At post-change, a higher percentage of students obtained sufficient sleep duration in the non-activity group (4.8%), but the effect size was not meaningful.

No differences in sleep-wake patterns or the percent of students obtaining sufficient sleep duration were found between students with or without before-school employment (Table 1). Across all 3 years, students with after-school employment had later bedtimes (6, 11, and 16 min, respectively), than non-employed students, with a small effect size found only at follow-up (*d* = 0.25). Wake time and sleep duration only differed at follow-up (7 min later and 10 min shorter), but neither effect size was meaningful. At follow-up, a higher percentage of students who were not employed obtained sufficient sleep duration (4.8%, Figure 2D), although the effect size was not meaningful.

## Discussion

This study adds to the literature on healthy school start times in three distinct ways by: (1) examining the impact of changing start times to include before- as well as after-school activities and employment, (2) examining differences in sleep-wake patterns between participating and non-participating students before and after a change in school start times, and (3) including elementary school students.

The only change in before-school activity participation was found for ES students, where participation decreased by ~50%. There are two primary reasons why this reduction was observed. First, with the later pre-change start time (9:00 a.m.), many students may have participated in activities as a way to support parents' needs to get to work. With the earlier post-change start time (8:00 a.m.), many families with working parents likely no longer needed this form of before-school child care. The change in before-school activities participation was similar to that seen in before-school childcare programs, where enrollment dropped 59% with earlier ES start times. The second reason for the decrease in before-school activities was elementary schools were instructed to not have any school-sponsored before-school activities. This policy was implemented by the district to prevent ES students from having to wake earlier than necessary. Participation in after-school activities remained the same across years for ES students.

For MS and HS students, no change was found in before-school activity participation or employment. Similar to the elementary school policy, middle and high schools were instructed not to add new before-school activities (activities that met before school prior to the start time change were allowed to remain before school, but no after-school activities were allowed to move to before school). This decision was to prevent a situation where the sleep benefits of a delayed secondary school start time would be negated by participation in before-school activities. As other districts consider or implement healthy secondary school start times, it is important for leaders to recognize the importance of ensuring that potential benefits of the new start times are not limited or lost by permitting school-sponsored activities before the first bell.

Later secondary school start times mean later school end times. In CCSD, middle school dismissal changed to 3:45 p.m., and high school dismissal moved to 3:30 p.m.. Not surprisingly, a small decrease in after-school activity participation was found, more so for MS students than HS students. However, while decreased participation in after-school activities is not a desirable outcome, the loss of after-school activity participation for about 3–4% of students should be viewed within the context of the significant increase in sleep duration for nearly all MS and HS students (*n* = ~18,000), in the district. The increase in sleep duration also benefitted students who continued to have before-school activities. It is also important to be aware that often there is an adjustment period when changing start times. In this study, it is reflected in the initial decrease, and then increase, in after-school employment, with a higher percent of students employed at follow-up than before the implementation of the later start time. Notably, the initial decrease was seen only among students who did not qualify for free/reduced lunch. Concerns about students' ability to work after school is often cited as a reason against delaying school start times, yet our findings show that if students need to work, that opportunity is not negatively impacted by a later dismissal time.

In terms of sleep-wake patterns, this study found that ES students who participated in after-school activities had earlier bedtimes, greater sleep duration, and a higher percent of students obtaining sufficient sleep. Although differences between groups were small, and effect sizes were negligible, these findings are noteworthy. This is because many elementary schools are required to start earlier as a result of implementing later secondary school start times. A common objection is the fear that ES students will have to sacrifice either sleep or after-school activities with earlier ES start times, but the findings reported here do not support those concerns.

For MS and HS students, differences in sleep-wake patterns between groups (participating vs. non-participating students) were found after the implementation of later start times. However, these differences were small and not clinically meaningful. On the other hand, at post-change and follow-up it was notable that even students with activities (before- or after-school), or those who were employed, had a clinically meaningful increase in sleep duration (~30–40 min) compared to pre-change/earlier start times. In addition, the percent of HS students who obtained sufficient sleep with later start times, regardless of activity or employment participation, was more than double that of the U.S. average (52.4–64.5% in current study vs. national average of 25.4%) (Kann et al., 2018). This finding alone reveals direct evidence of a health benefit due to the later start times.

Although this study included data from before and after the implementation of new start times, surveys were anonymous, thus data were not able to be linked longitudinally. Additional study limitations include the self-reporting of both sleep and activity/employment participation. Future studies should include objective measures of sleep (e.g., actigraphy). In addition, the pre-change survey was conducted in April/May (>4 weeks after the start of daylight saving time) due to the district's decision to change start times occurring shortly before the end of the school year, while the post-change and follow-up surveys were conducted in late February/early March (prior to the change to daylight saving time); thus, participation in some activities may have been affected by seasonal differences. However, it is important to note that activities and employment that were queried for this study were non-seasonal. On the other hand, participation in athletics varies by season, with a different number of sports competing in the winter vs. the spring. Thus, we did not include athletic participation data in this study. Finally, the CCSD is a suburban school district, and thus results may not be generalizable to urban or rural school districts.

These novel findings highlight that small changes to before- and after-school non-athletic activity participation may be anticipated for school districts implementing later middle and high school start times. While consistent with concerns that have been raised about changing school start times (Owens, 2014; Fitzpatrick et al., 2020), the small changes in participation also need to be considered within the context of the significant increases in student sleep duration, which has short- and long-term benefits physical, mental, and academic health (Owens, 2014).

Finally, it is important to note that structural mandates, such as the district's decision not to allow any new before-school sponsored activities for middle and high schools, and not to permit any sponsored before-school activities for elementary schools, are also important to consider as part of this policy change.

## Data availability statement

The raw data supporting the conclusions of this article will be made available by the authors, without undue reservation.

## Ethics statement

The studies involving human participants were reviewed and approved by Cherry Creek School District Research Review Committee. Written informed consent for participation was not provided by the participants' legal guardians/next of kin because the study included an anonymous survey. However, parents had the option to opt their student out of participation. Students were information that participation was voluntary, they could stop being in the study at any point, and there was no penalty if they chose not to participate.

## Author contributions

LM conducted the data analyses and prepared the manuscript. All authors contributed to the conceptualization and design of the study, the acquisition and interpretation of the data, and conceptualization of the manuscript, as well as editing and approving the submitted version. LM had full access to all data for the study and had the final responsibility for the decision to submit for publication.

## Funding

This study was supported by the Robert Wood Johnson's Evidence for Action program (Grant #75277). The funder had no role in the design and conduct of the study; collection, management, analysis, and interpretation of the data; preparation, review, or approval of the manuscript; and decision to submit the manuscript for publication.

## Conflict of interest

The authors declare that the research was conducted in the absence of any commercial or financial relationships that could be construed as a potential conflict of interest.

## Publisher's note

All claims expressed in this article are solely those of the authors and do not necessarily represent those of their affiliated organizations, or those of the publisher, the editors and the reviewers. Any product that may be evaluated in this article, or claim that may be made by its manufacturer, is not guaranteed or endorsed by the publisher.

## Author disclaimer

The study does not necessarily reflect the opinions or views of the Robert Wood Johnson Foundation.
