# Supplementary material for: Changing school start times: Impact on extracurricular activities and employment
Source: Front Sleep. 2022 Nov 2;1:1044457. doi: 10.3389/frsle.2022.1044457 (PMC12713866; doi:10.3389/frsle.2022.1044457)
Supplement: Supplementary file 1 [file Data_Sheet_1.docx]

**Changing School Start Times: Impact on Extracurricular Activities and Employment**

**Online Supplement**

Table S1. Demographic characteristics and sleep-wake patterns of all study participants.

|  | **Elementary School**  **(Grades 3-5)** | | |  | **Middle School**  **(Grades 6-8)** | | |  | **High School**  **(Grades 9-12)** | | |
| --- | --- | --- | --- | --- | --- | --- | --- | --- | --- | --- | --- |
|  | Pre-Change | Post-Change | Follow-Up |  | Pre-Change | Post-Change | Follow-Up |  | Pre-Change ^a^ | Post-Change | Follow-Up |
| Total N | 8,349 | 7,943 | 8,253 |  | 7,531 | 8,435 | 8,889 |  | 5,640 | 7,416 | 9,783 |
| % Enrolled | 66.9 | 62.6 | 66.1 |  | 59.2 | 65.3 | 68.4 |  | 45.0 | 43.5 | 56.1 |
| % Female | 48.4 | 50.0 | 49.4 |  | 51.9 | 50.9 | 50.0 |  | 52.3 | 52.5 | 51.7 |
| % FRL | 20.5 | 24.2 | 16.9 |  | 18.8 | 23.0 | 16.9 |  | 17.4 | 21.1 | 14.9 |
| Race/Ethnicity |  |  |  |  |  |  |  |  |  |  |  |
| % White | 59.8 | 57.9 | 54.1 |  | 60.0 | 58.0 | 54.8 |  | 58.3 | 57.2 | 53.0 |
| % Black | 8.8 | 8.8 | 8.8 |  | 9.3 | 10.3 | 9.5 |  | 10.6 | 10.4 | 10.7 |
| % Hispanic | 15.3 | 15.8 | 19.6 |  | 14.8 | 16.1 | 19.5 |  | 15.3 | 16.3 | 19.8 |
| % Asian | 8.1 | 8.5 | 8.6 |  | 9.0 | 8.5 | 9.0 |  | 9.5 | 9.5 | 9.9 |
| % MR/AIAN/NHOPI | 8.0 | 8.9 | 8.8 |  | 6.9 | 7.1 | 7.2 |  | 6.3 | 6.6 | 6.7 |
| Sleep-Wake Patterns |  |  |  |  |  |  |  |  |  |  |  |
| Mean Bedtime (SD) | 21:12 (0:57) | 21:01 (0:58) | 21:01 (0:58) |  | 21:49 (0:57) | 21:58 (1:01) | 21:58 (1:01) |  | 22:23 (0:59) | 22:35 (1:02) | 22:46 (1:05) |
| Mean Wake Time (SD) | 7:05 (0:51) | 6:42 (0:46) | 6:42 (0:51) |  | 6:27 (0:39) | 7:05 (0:42) | 7:02 (0:42) |  | 5:46 (0:42) | 6:44 (0:41) | 6:45 (0:42) |
| Mean Sleep Duration (SD) [hours] | 9.87 (1.12) | 9.69 (1.07) | 9.67 (1.08) |  | 8.63 (1.02) | 9.10 (1.07) | 9.06 (1.08) |  | 7.38 (1.08) | 8.15 (1.12) | 7.99 (1.12) |

^a^Pre-change high school surveys included students in grades 9-11, as the pre-change cohort of 12^th^ grade students would have graduated by the time the post-change survey was administered

FRL: Free or Reduced Lunch Status

MR/AIAN/NHOPI: Mixed Race/American Indian or Alaskan Native/Native Hawaiian or Other Pacific Islander

Table S2. Demographic characteristics of students who participate in before school and after school extracurricular activities.

|  | **Elementary School**  **(Grades 3-5)** | | |  | **Middle School**  **(Grades 6-8)** | | |  | **High School**  **(Grades 9-12)** | | |
| --- | --- | --- | --- | --- | --- | --- | --- | --- | --- | --- | --- |
|  | Pre-Change | Post-Change | Follow-Up |  | Pre-Change | Post-Change | Follow-Up |  | Pre-Change ^a^ | Post-Change | Follow-Up |
| Before School % Participated | 30.5 | 14.4 | 14.0 |  | 11.0 | 11.6 | 10.7 |  | 11.1 | 12.7 | 12.7 |
| Gender |  |  |  |  |  |  |  |  |  |  |  |
| % Male | 28.0 | 14.7 | 14.8 |  | 11.1 | 11.5 | 10.3 |  | 11.4 | 11.7 | 11.1 |
| % Female | 33.6 | 13.5 | 13.2 |  | 10.4 | 11.4 | 11.1 |  | 10.9 | 14.2 | 14.3 |
| FRL |  |  |  |  |  |  |  |  |  |  |  |
| % No | 30.7 | 13.5 | 13.5 |  | 10.4 | 11.4 | 10.8 |  | 11.2 | 13.4 | 13.1 |
| % Yes | 29.4 | 17.0 | 16.4 |  | 13.3 | 12.0 | 10.1 |  | 10.7 | 10.3 | 10.4 |
| Race/Ethnicity |  |  |  |  |  |  |  |  |  |  |  |
| % White | 31.1 | 12.5 | 12.3 |  | 9.8 | 11.5 | 11.0 |  | 11.6 | 14.1 | 13.6 |
| % Black | 32.2 | 21.9 | 19.8 |  | 14.6 | 12.4 | 12.8 |  | 10.0 | 11.7 | 12.8 |
| % Hispanic | 29.6 | 14.2 | 15.8 |  | 11.7 | 9.9 | 9.2 |  | 9.1 | 9.3 | 9.3 |
| % Asian | 29.8 | 15.9 | 14.0 |  | 11.4 | 14.1 | 11.4 |  | 11.7 | 14.6 | 14.3 |
| % MR/AIAN/NHOPI | 29.4 | 15.0 | 15.1 |  | 10.6 | 10.0 | 8.1 |  | 12.6 | 12.5 | 13.9 |
| After School % Participated | 58.3 | 59.3 | 57.9 |  | 62.6 | 58.3 | 54.0 |  | 56.0 | 52.8 | 50.3 |
| Gender |  |  |  |  |  |  |  |  |  |  |  |
| % Male | 52.4 | 51.0 | 50.3 |  | 56.8 | 52.9 | 47.6 |  | 50.4 | 46.8 | 43.7 |
| % Female | 65.1 | 67.9 | 65.6 |  | 69.9 | 64.7 | 60.5 |  | 63.4 | 59.4 | 56.4 |
| FRL |  |  |  |  |  |  |  |  |  |  |  |
| % No | 60.7 | 62.3 | 59.6 |  | 65.1 | 62.1 | 56.6 |  | 58.1 | 55.3 | 51.4 |
| % Yes | 49.3 | 49.9 | 49.7 |  | 51.8 | 45.7 | 41.2 |  | 46.3 | 43.5 | 43.9 |
| Race/Ethnicity |  |  |  |  |  |  |  |  |  |  |  |
| % White | 60.0 | 61.5 | 61.0 |  | 65.9 | 62.3 | 59.0 |  | 56.8 | 55.9 | 52.4 |
| % Black | 54.2 | 53.7 | 50.4 |  | 58.2 | 50.3 | 44.0 |  | 53.9 | 46.1 | 48.5 |
| % Hispanic | 51.9 | 53.1 | 49.0 |  | 55.3 | 47.5 | 43.0 |  | 49.9 | 45.7 | 41.2 |
| % Asian | 65.0 | 66.2 | 67.5 |  | 71.2 | 70.9 | 59.1 |  | 65.6 | 62.7 | 60.2 |
| % MR/AIAN/NHOPI | 59.0 | 56.3 | 56.7 |  | 58.4 | 55.0 | 53.1 |  | 52.6 | 49.0 | 48.3 |

^a^Pre-change high school surveys included students in grades 9-11

FRL: Free or Reduced Lunch Status

MR/AIAN/NHOPI: Mixed Race/American Indian or Alaskan Native/Native Hawaiian or Other Pacific Islander

Table S3. Demographic characteristics of students who were employed before and after school.

|  | **High School**  **(Grades 9-12)** | | |
| --- | --- | --- | --- |
|  | Pre-Change ^a^ | Post-Change | Follow-Up |
| Before School % Employed | 1.3 | 1.5 | 2.1 |
| Gender |  |  |  |
| % Male | 1.6 | 1.5 | 2.6 |
| % Female | 0.8 | 1.4 | 1.6 |
| FRL |  |  |  |
| % No | 1.2 | 1.5 | 2.0 |
| % Yes | 1.6 | 1.4 | 2.5 |
| Race/Ethnicity |  |  |  |
| % White | 1.1 | 1.3 | 1.7 |
| % Black | 1.2 | 1.3 | 3.5 |
| % Hispanic | 1.6 | 1.9 | 2.4 |
| % Asian | 0.7 | 1.6 | 1.8 |
| % MR/AIAN/NHOPI | 1.0 | 1.4 | 2.8 |
| After School % Employed | 19.9 | 16.9 | 24.3 |
| Gender |  |  |  |
| % Male | 18.4 | 16.0 | 22.4 |
| % Female | 19.4 | 17.8 | 26.0 |
| FRL |  |  |  |
| % No | 20.5 | 17.0 | 23.9 |
| % Yes | 16.9 | 16.7 | 26.3 |
| Race/Ethnicity |  |  |  |
| % White | 21.2 | 18.9 | 26.1 |
| % Black | 15.8 | 14.1 | 21.3 |
| % Hispanic | 18.3 | 15.6 | 25.5 |
| % Asian | 10.1 | 11.9 | 15.2 |
| % MR/AIAN/NHOPI | 17.6 | 14.9 | 24.1 |

^a^Pre-change high school surveys included students in grades 9-11

FRL: Free or Reduced Lunch Status

MR/AIAN/NHOPI: Mixed Race/American Indian or Alaskan Native/Native Hawaiian or Other Pacific Islander
